# Impact of epicardial ablation of concomitant atrial fibrillation on atrial natriuretic peptide levels and atrial function in 6 months follow-up: does preoperative ANP level predict outcome of ablation?

**DOI:** 10.1186/1749-8090-8-218

**Published:** 2013-11-28

**Authors:** Marek Pizon, Norbert Friedel, Monika Pizon, Miriam Freundt, Michael Weyand, Richard Feyrer

**Affiliations:** 1 Department of Cardiac Surgery, Clinic of Bayreuth, Preuschwitzerstr 101, 95455 Bayreuth, Germany; 2 Transfusion Center Bayreuth, Kurpromenade 2, 95448 Bayreuth, Germany; 3 Center of Cardiac Surgery, University Hospital of Erlangen, Krankenhausstr 12, 91054 Erlangen, Germany; 4 Rochester General Hospital, 1425 Portland Ave Rochester, Rochester 14621, NY, USA

**Keywords:** Atrial fibrillation, Cardiac surgery, Atrial natriuretic peptide, Surgical ablation, Epicardial ablation, HIFU

## Abstract

**Background:**

Epicardial ablation concomitant to cardiac surgery is an easy and safe approach to treat atrial fibrillation (AF), but its efficacy in longstanding persistent (LsPe) AF remains intermediate. Although larger left atrial size has been associated with worse outcome after ablation, biochemical predictors of success are not well established. The aim of this study was to evaluate relationship between biochemical marker, echo-characteristic and cardiac rhythm in 6 months follow-up after epicardial ultrasound (HIFU) ablation.

**Methods:**

We included 78 consecutive patients, who underwent elective cardiac surgery. 42 patients with AF (11.9% paroxysmal, 23.8% persistent, 64.3% LsPeAF) underwent concomitant HIFU ablation (AF ablation group), 16 with AF underwent cardiac surgery without ablation (AF control) and 20 had preoperatively normal sinus rhythm (SR control). We measured plasma ANP secretion before, on postoperative day (POD) 1, POD 7 as well as 3 and 6 months after surgery. Moreover, we estimated cardiac rhythm and atrial mechanical function by Atrial Filling Fraction (AFF) and A-wave velocity in follow-up.

**Results:**

Baseline ANP levels were higher in patients with LsPeAF, as compared to the paroxysmal and permanent AF and to the SR control group. Patients with LsPeAF (n = 27) who converted to SR had preoperatively smaller left atrial diameter (LAD) and LA area (p < 0.05) and higher ANP level (p = 0.009) than those who remained in AF at 6 months after ablation. Multivariate regression analysis revealed that only preoperative ANP level was an independent predictor of cardiac rhythm after ablation. Patients with LsPeAF and preoperative ANP >7.5 nmol/l presented with SR in 80%, in contrast to those with ANP <7.5 nmol/l who converted to SR in 20%. We detected gradual increase of AFF and A-velocity at 6 months after ablation (p < 0.05) solely in AF ablation group. ANP levels were increased on POD 1 in ablation group (p < 0.05), without changes in further follow-up.

**Conclusion:**

Our results indicate that preoperative ANP levels may be a new biochemical predictor of successful epicardial ablation in patients with concomitant LsPeAF. HIFU ablation caused a significant improvement of atrial mechanical function and gradual increase of AFF and did not associate with alteration of atrial endocrine secretion at 6 months follow-up.

## Background

Atrial fibrillation (AF) is the most common supraventricular cardiac arrhythmia, which affects approximately 6–10.2 million people in Europe, 2.2 million Americans and its prevalence increases with age [1,2]. Affected patients have a five times bigger stroke risk and twice the risk of death. AF reduces left ventricular (LV) function, diminishes exercise tolerance, cognitive capacity, impairs quality of life (QoL) as well as elevates atrial natriuretic peptide (ANP) secretion [3-5]. In cardiac surgery AF is associated with increased risk of perioperative death and decreased short- and long-term survival [6].

AF is classified as paroxysmal (Pa; self terminating within 7 days or cardioverted ≤48 hours), persistent (Pe; sustained >7 days or cardioverted after 48 hours but prior to 7 days), longstanding persistent (LsPe; continuous >1 year) and permanent (continuous >1 year, presence of AF is accepted and the therapy does not affect the restoration of sinus rhythm by any means) [7].

Current guidelines recommend concomitant surgical ablation in patients with AF who undergo cardiac surgery [1,7]. The procedure consists of formation of electrical conduction blocks by scalpel like in classic Cox-maze procedure (CMP) or an alternative source of energy such as high intensity focused ultrasound (HIFU), radiofrequency (RF), cryoablation, microwave and laser. The aim is to isolate trigger foci and inhibit macro-reentry circuits, essential in pathogenesis of AF. HIFU allows epicardial ablation on beating heart and its safety and efficacy is well-known [8-12]. The primary goal of ablation is to restore the active function of the atria, to improve cardiac hemodynamics and reduce the risk of embolism. The success of surgical ablation depends on type and duration of AF and left atrium (LA) size [13,14]. Patients with LsPe AF have the most enlarged atria and show generally the poorest outcome after ablation [11,12].

Yoshihara et al. revealed that atrial natriuretic peptide (ANP) might be a potential predictive marker of heart rhythm after ablation by maze procedure [15]. ANP belongs to the group of natriuretic homeostatic peptides. It increases diuresis and natriuresis, stimulates vasodilatation, inhibits the renin-angiotensin-aldosteron system (RAAS), the sympathetic nervous system and regulates water balance. ANP is synthesized in atrial myocytes and the key stimulus for its secretion in AF is the stretch of atrial wall (rather than atrial pressure) [16].

Classic CMP has been reported to cause a dramatic decrease of ANP levels for at least 7 days after procedure, leading to post-operative severe fluid retention, oedema and recurrent pleural effusion [17]. To our knowledge, the effect of HIFU on post-operative plasma ANP levels has not yet been studied. The biochemical predictors of cardiac rhythm after epicardial ablation are not yet known. The aims of our prospective study were: (1) to evaluate whether preoperative ANP level might have a predictive value for the success rate of epicardial HIFU ablation (2) to estimate the recovery of atrial mechanical function following epicardial HIFU ablation and their impact on alteration of endocrine secretion in 6 moths follow-up (3) to analyze the effect of epicardial HIFU ablation of concomitant AF on atrial endocrine secretion based on serial postoperative ANP levels.

## Methods

### Patients’ population

We conducted a prospective trial with ‘all-comers’ design and included 78 consecutive patients, who underwent elective cardiac surgery from 2011 to 2012. 42 patients with AF due to organic heart disease underwent epicardial HIFU ablation concomitant to cardiac surgery (AF ablation group). 16 patients with AF due to organic heart disease underwent cardiac surgery without ablation (AF control group without ablation). 20 patients underwent cardiac surgery and had normal sinus rhythm (SR control group). Detailed baseline and operative characteristics of our study population are presented in Table 1. Ablation was done in all-comers design, i.e. patients were not selected in terms of type and duration of AF, LA size and other parameters important for treatment efficacy. Our study had an “intention to treat”, i.e. ablation was offered to every patients affected with AF. Therefore AF control group without ablation included only patients, in whom we found intraoperative obstacles to safely perform the procedure. Those were adhesions in re-operated patients or after radiotherapy (breast cancer), chronic constrictive pericarditis, left atrial myxomas (because of embolism risk at manipulation), endocarditis with aortic root abscess extending into the dome of left atrium. One patient had dislodged left atrial apandage occluder and other patient, with accepted permanent AF for many years by himself and his cardiologist, denied ablation, but agreed to cardiac surgery. The study was approved by the research ethics committee of Friedrich-Alexander University Erlangen-Nürnberg in Germany, and all patients had given informed consent preoperatively.

**Table 1:** Basal and operative characteristics of patients enrolled into the study

	***AF ablation group (n = 42)***		***AF control group without ablation (n = 16)***		***SR control group (n = 20)***	***p-value***
**Demography**						
Age [years]	71.3 ± 8.3		73.8 ± 6.6		68.9 ± 7.0	0.16
Male gender, n (%)	31 (73,8%)		12 (75%)		15 (75%)	0.99
Body Mass Index (kg/m^2^)	29.4 (5.3)		27.5 (4.9)		29.2 (3.9)	0.38
**AF duration [months]**	64.8 ± 57.1		73.0 ± 92.9		0	0.29^1^
**Type of AF %, (n)**						
**HRS/AHA (ACC:**	initial	reclassified	initial	reclassified		
1. Paroxysmal	11.9%	**11.9% (5)**	25.0%	**25.0% (4)**	0	
2. Persistent	23.8%	**23.8% (10)**	31.2%	**31.2% (5)**	0	
3. Longstanding pers.	21.4%	**64.3% (27)**	12.5%	**43.7% (7)**	0	
4. Permanent	42.9%	------	31.2%	------	0	
**Cox - classification**:						
Intermittent	**35.7%** (15)		**56.2%** (9)		0	0.23^1^
Continuous	**64.3%** (27)		**43.7%** (7)		0	0.23^1^
**Preop. cardioversion (DC), n (%)**	11 (26.2%)		5 (31.2%)		1 (5%)	
**Preop. catheter ablation, n (%)**	2 (4.8%)		0		0	
**Preop. perm. anticoagulation, n (%)**	26 (61.9%)		9 (56.2%)		1 (5%)	
**Previous cardiac surgery**	0		3 (18.75%)		0	
**Comorbidity, n (%)**						
Stroke	6 (14.3%)		2 (12.5%)		1 (5%)	
Heart failure (CHF)	25 (59.5%)		10 (62.5%)		7 (35%)	
Myocardial infarction	12 (28.6%)		5 (31.3%)		7 (35%)	
Pre-op. creatinine [mg/dl]	1.3 ± 0.32		1.29 ± 0.35		1.09 ± 0.28	0.13
**ECHO data**						
EF (Simpson) [%]	54.1 ± 10.8		54.1 ± 14.4		61.0 ± 9.0	0.07
Max. LA area [cm2]	**27.7 (24.1-32.4)**		**27.8 (23.5-30.4)**		**20.3 (14.7-24.6)**	0.65^1^**, p < 0.001**^**2**^
Max. LA diameter (LAD) [mm]	**48.5 ± 7.0**		**47.5 ± 7.2**		**41.7 ± 6.5**	0.65^1^**, p < 0.01**^**2**^
LA AFF [%]	**0 (0–25.2)**		**0 (0–34.9)**		**43.2 (32.4-52.8)**	p > 0.05^1^**, p < 0.001**^**2**^
**Procedures**						
**Without mitral-surg., n (%)**	**32 (76%)**		**12 (75%)**		**18 (90%)**	1.0^1^,^2^0.37^2^
CABG	12 (28.6%)		2 (12.5%)		13 (65%)	
AVR	7 (16.7%)		3 (18.75%)		5 (25%)	
Aortic replacement	1 (2.3%)		-		-	
AVR + CABG	11 (26.2%)		3 (18.75%)		-	
AVR + Aortic replacement	-		2 (12.5%)			
Aortic replacement + CABG	1 (2.3%)				-	
Tricuspid valve surgery	-		1 (6.25%)		-	
VSD	-		1 (6.25%)		-	
**With mitral-surgery, n (%)**	**10 (23.8%)**		**4 (25%)**		**2 (10%)**	1.0^1^, 0.37^2^
**Euroscore II (%)**	**3.0 ± 2.36**		**4.3 ± 3.15**		**1.94 ± 1.54**	**0.007**^**2**^
**LAA Amputation, n (%)**	9 (21.4%)		2 (12.5%)		1 (5%)	
**RAA Amputation, n (%)**	42 (100%)		16 (100%)		20 (100%)	

*Abbreviations comment*: *AF* atrial fibrillation, *SR* sinus rhythm, *LA* left atrium, *AFF* atrial filling fraction, *AVR* aortic valve replacement, *VSD* ventricular septal defect, *MV* mitral valve, *MVR* mitral valve replacement, *LAA* left atrial appendage, *RAA* right atrial appendage; ^1^represents the differences between AF ablation group and AF control group without ablation; ^2^represents the differences between AF ablation group vs SR control group and AF control group without ablation vs SR control group. Values are demonstrated as mean ± SD or median (25–75 percentiles).

AF type was classified according to current ACC/AHA/ESC guidelines: paroxysmal (Pa), persistent (Pe), longstanding persistent (LsPe) and permanent. According to the guidelines, the term permanent AF refers to patients for whom a decision has been made not to restore SR by any means, including catheter or surgical ablation. Because in our study every patient with AF was considered for surgical ablation, patients previously classified as having permanent AF are in the following referred to as LsPe AF [8].

Exclusion criteria included significant systolic dysfunction of the left ventricle with an ejection fraction (EF) <35%, renal insufficiency with preoperative creatinine >2.0 mg/dl and hyperthyroidism (TSH <0.3 μU/ml, fT4 >1.65 ng/ml, fT3 >4.0 pg/ml), as factors with a potential effect on ANP levels.

### Surgical technique and ablation procedure

All patients underwent median sternotomy, heparinization, amputation of the right atrial appendage (RAA), institution of extracorporeal circulation and cardioplegic cardiac arrest following institutional protocol. For epicardial HIFU ablation we used the Epicor system (St Jude Medical). The procedure occurred following full heparinization and before institution of cardiopulmonary bypass. The left atrial (LA) size was measured to select the appropriate transducer and Ultra Cinch was positioned through the transverse sinus. On beating heart the posterior wall of left atrium around the pulmonary veins was isolated in 10-minutes algorithm, creating so-called box lesion. In addition mitral line lesion was created with the Ultra Wand (applied for 2 mins) in all patients undergoing ablation.

### Post-ablation treatment

Patients, who had undergone ablation, received a bolus of 300 mg Amiodarone intraoperatively, followed by continuous intravenous infusion for 24–72 hours and oral therapy for at least 3 months. 3 months postoperatively the heart rhythm was reevaluated and amiodarone therapy was stopped in patients with sinus rhythm or continued in patients with ongoing AF. Maximal duration of amiodarone therapy was 6 months. If contraindications or bradycardia occurred, amiodarone therapy was interrupted or continued with reduced dosage. Anticoagulation was initiated 8 hours postoperatively with continuous intravenous heparin infusion. On post-op day (POD) 2–3 the heparin drip was switched to oral Phenprocoumon with a goal INR of 2.0-2.5. Patients remained anticoagulated for at least 6 months postoperatively.

### Measurement of plasma proANP (1–98)

For proANP (1–98) measurement peripheral blood was collected 1 day preoperatively, and POD 1 and 7, as well as 3 and 6 months after surgery. Blood was drawn following 20 minutes rest in supine position.

Blood samples were collected into EDTA tubes, placed on ice, centrifuged at +4°C (40°F) within one hour and the plasma was stored as aliquots at -80°C (-112°F) until analyzed. The quantitative determination of plasma proANP (1–98) concentration was performed using an ELISA kit (Biomedica, Austria) following the manufacturer’s directions. The lower detection limit for pro ANP (1–98) was 0.05 nmol/l and the intra- and inter-assay of CV % were 2% and 4%, respectively.

### Follow- up and echocardiography

Patients were seen at 3 and 6 months follow-up in our clinic. The encounter included physical examination, blood collection for ANP, ECG and transthoracic echocardiogram (TTE). At 6 months follow-up additionally a 24 hour-ECG (Holter) monitoring was recorded. TTEs were performed by standardized institutional protocol, using Vivid 7 (General Electric, GE) and read by the same experienced physician to minimize inter-rater variability. In addition to standard evaluation of left ventricular function, LA size was measured and hemodynamic function of both atria was assessed. EF was measured by Simpson method in two projections. Planimetric measurement of area and diameters (transverse and longitudinal) of the left atrium was assessed in apical four-chamber view. For analysis of results the maximal transverse diameter of LA (LAD) and maximal LA area (LA area) were used. Mechanical function of the atria was analyzed in Pulsed-Doppler (PW) with measurement of trans-mitral and trans-tricuspid inflow from left and right ventricle, respectively. Velocity of A wave on the level of mitral and tricuspid valve rings and Atrial Filling Fraction (AFF) for the left atrium were measured on the basis of average values from 3 consecutive cardiac cycles. AFF reflects the active contribution of the LA to diastolic filling of the left ventricle (LV) and was calculated from percentage ratio of velocity time integral (VTI) for A wave to VTI of total diastolic inflow.

Complete follow-up in the groups was done in 90.5%, 93.8% and 100% of patients (92,9%,100%,100% of survivors) in the AF ablation group, AF control group without ablation and SR control group, respectively.

### Statistical analysis

All data are expressed as the means (±SD) or medians (25–75 percentiles). Clinical data were analyzed by unpaired Student *t*-test or the Mann–Whitney test. ProANP (1–98) kinetic were evaluated by 1-way ANOVA or ANOVA on ranks. *Chi*-Square test or Fisher’s exact test was used to compare categorical variables between groups.

We tested potential preoperative predictors of ablation outcome in a three steps analysis. First, the impact on binary ablation outcome (SR or AF) at 6 months of each isolated parameter was studied in an univariate manner using \Fischer exact test for 2 × 2 contingency tables. Second, a multiple regression analysis was used to identify independent predictors of cardiac rhythm after ablation. Thereafter, the quality and power of discrimination was estimated by calculation of the area under the receiver operating characteristic (ROC) curve. Cut-off values of predictors were calculated for the best sensitivityand specificity of tests. Assessment of Goodness of Fit (GOF) for logistic regression model was ascertained by the Hosmer-Lemeshow method [18].

p <0.05 was considered as significant. Statistical analyses were performed with SigmaPlot 11.0 for Windows (Systat Software GmbH, Germany).

## Results

### Study groups and baseline clinical characteristics

Baseline characteristics are shown in Table 1. LAD before surgery was found increased according to the total burden of AF: being lowest in SR control group (n = 20), increased in Pa and Pe AF (n = 15) and largest in LsPe AF (n = 27) (mean 41.7 ± 6.5 vs 46.2 ± 6.5 vs 49.7 ± 7.0 mm). Furthermore, in the group with LsPe AF LAD was statistically significant larger than in SR control group (p < 0.001). LA areas expressed a similar pattern before the procedure, 20.3 cm^2^ (14.7-24.6) in SR control group, 24.6 cm^2^ (22.3-27.5) in Pa and Pe AF and 28.0 cm^2^ (26.6-34.0) in LsPe AF. Solely in the group with LsPe AF LA area was significantly larger in comparison to SR controls (p < 0.001).

### Results of ablation

In the AF ablation group (n = 42) no periprocedural complications were observed. No death occurred within 30-days postoperatively. Two patients required pacemaker implantation (4.7%) due to sick sinus syndrome and chronotropic incompetence in 6 months follow-up.

Sinus rhythm (SR) was found in 66.7%/100% patients with Pa AF, 100%/90% with Pe AF and in 33.3%/56% with LsPe AF at 3 and 6 months. 68.4% of the patients achieved freedom from AF and flutter within 6 months, 92.8% of patients with Pa and Pe, and 56% of patients with LsPe AF. In AF control group without ablation SR conversion rate was 0%/25% at 3 and 6 months after cardiac surgery, respectively.

In patients with SR at 6 months after ablation, 84.6% (22/26) and 88.5% (23/26) had an atrial-filling wave (A-wave) in trans-mitral and trans-tricuspid inflow, respectively, and both-side A waves were seen in 80.8% of patients (21/26). A total lack of A wave was found in 2 patients with LsPe AF.

**Figure 1:**
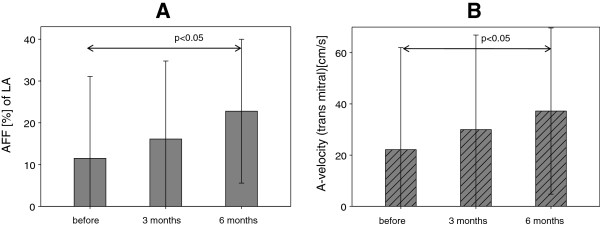
**Atrial mechanical function after ablation. (A)** Atrial Filling Fraction (AFF) and **(B)** A-velocity of left atrium before, 3 and 6 months after HIFU ablation.

In AF ablation group we observed significantly improved atrial mechanical function in follow-up. We noted increased velocity of left-side A wave and AFF at 6 months, compared to the preoperative values (mean: 22.1 ± 39 and 11.5 ± 19.5 before ablation vs 37.2 ± 32.4 cm/s and 22.8 ± 17.1% after 6 months for A wave and AFF, respectively, p < 0.05) (Figure 1). In contrast, A-velocity and AFF did not show significant changes in AF control group without ablation and in SR control group.

### Preoperative LAD and LA area as predictors of ablation outcome

**Figure 2:**
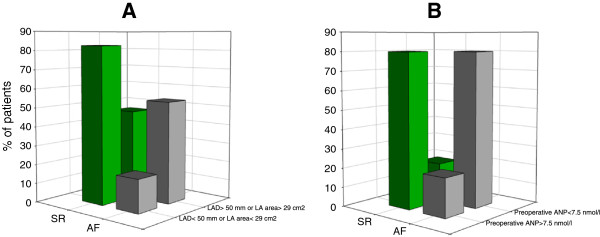
**Impact of preoperative ANP, LAD and LA area on conversion rate at 6 months after ablation. (A)** LA area and LAD in paroxysmal, persistent and longstanding persistent AF **(B)** preoperative plasma ANP level in longstanding persistent AF; ANP: atrial natriuretic peptide; LAD: left atrial diameter; LA: left atrium.

In AF ablation group patients who successfully converted to SR at 6 months (n = 26) had preoperatively smaller LAD (p = 0.01) and LA area (p = 0.02) than those who remained in AF (n = 12). The cut-off point for LAD was 50 mm, and LA area 29 cm^2^ (sensitivity 76.9%, specificity 66.6% and positive predictive value 83.3%). In patients with LAD ≤ 50 mm and LA area ≤ 29 cm^2^ (n = 24), effectiveness of ablation in restoring SR was 83.3% at 6 months and it was significantly higher compared to the group with LAD > 50 mm and LA area > 29 cm^2^ (n = 14), in which it was only 42.8% (p = 0.03) (Figure 2).

### Kinetics of plasma ANP

**Figure 3:**
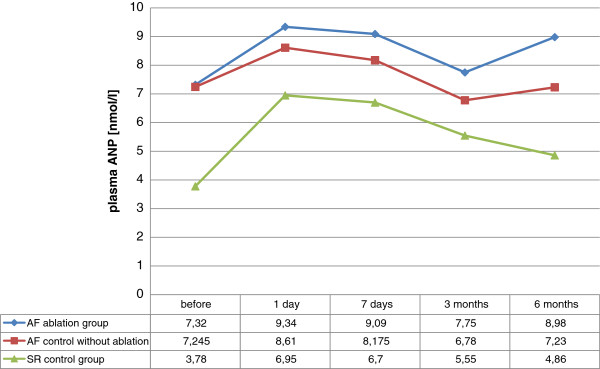
**Kinetics of plasma ANP in the study groups.** Values are demonstrated as median.

Plasma ANP level changes in each group are shown in Figure 3. In the AF ablation group ANP levels increased significantly on POD 1 compared to the baseline (p = 0.04) and did not show significant changes in further follow-up. In the AF control group without ablation ANP levels did not change significantly. However, in the SR control group ANP levels increased significantly within 7 days, then gradually decreased, reaching significant decrease 6 months after the procedure. ANP concentrations did not significantly differ between the AF ablation group and the AF control group without ablation during follow-up. ANP levels in the AF ablation group were higher compared to the SR control group, preoperatively (median 7.23 vs 3.78 nmol/l, p = 0.001), as well as on POD 1 (median 9.34 vs 6.95 nmol/l, p = 0.04), 3 months (median 7.75 vs 5.55 nmol/l, p = 0.03) and 6 months postoperatively (median 8.98 vs 4.86 nmol/l, p = 0.001). Whereas, in the AF control group without ablation plasma ANP levels were higher in comparison to the SR control group, preoperatively (median 7.24 vs 3.78 nmol/l, p = 0.03) and at 6 months follow-up (median 7.23 vs 4.86 nmol/l, p = 0.01).

### Plasma ANP at baseline

**Figure 4:**
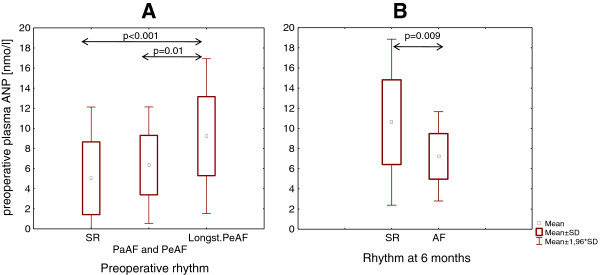
**Preoperative ANP. (A)** Comparison of the groups with longstanding persistent AF (Longst.Pe AF, n = 27), paroxysmal (PaAF) and persistent (PeAF) AF (n = 15) and SR control group (n = 20), **(B)** Comparison of individuals according to rhythm at 6 months after ablation in longstanding persistent AF. ANP: atrial natriuretic peptide.

Preoperative plasma ANP levels were considered as baseline values. They were significantly higher in patients with AF (n = 58), as compared to individuals with SR (n = 20) (p = 0.01). As shown in Figure 4, baseline ANP concentrations were found with increasing levels according to the total burden of AF, with being lowest in the SR control group (n = 20), increased in patients with Pa and Pe AF (n = 15) and highest in patients with LsPe AF (n = 27). Patients with LsPe AF had significantly higher baseline ANP levels, as compared to the patients with Pa and Pe AF (mean 9.22 ± 4.0 vs 6.4 ± 2.7 nmol/l, p = 0.01) and to the SR control group (mean 9.22 ± 4.0 vs 5.04 ± 3.6 nmol/l, p < 0.001).

In AF ablation group among individuals, who had SR after 6 months (n = 26), we observed higher ANP concentrations before the operation and in follow-up, as compared to the patients who remained in AF despite ablation (n = 12), but the differences were not statistically significant.

Whereas in patients with LsPe AF (n = 27) we found significantly higher preoperative plasma ANP levels in those individuals, who had converted to SR after 6 months, as compared to those who remained in AF (median 9.95 [7.53-14.02] vs 6.86 [5.68-8.06] nmol/l, p = 0.009) (Figure 4).

### Preoperative ANP levels as predictor of cardiac rhythm after ablation

To determine predictors of cardiac rhythm after HIFU ablation in patients with LsPe AF (n = 27), we compared preoperative variables between those individuals, who had successfully converted to SR (n = 14) and those who remained in AF (n = 11) at 6 months after procedure. Preoperative ANP levels, LAD and LA area were significantly higher, smaller and lower respectively in individuals with SR than in those with AF at 6 months after ablation (Table 2). Whereas preoperative duration of AF, age and gender did not significant differ.

**Table 2:** Preoperative characteristics of patients with longstanding persistent AF

	**AF at 6 months**	**SR at 6 months**	**p value**
**Age (yrs)**	78.0 (66.25-79.25)	74.5 (62.5-80.0)	0.87
**Gender (male/female)**	8/3	11/3	1.0
**Duration of AF (yrs)**	8.0 (3–10)	5.0 (3–9,5)	0.39
**LAD (preop.) [mm]**	53.1 ± 6.2	47.1 ± 6.2	**0.01**
**LA area (preop.) [cm**^**2**^**]**	33.95 (27.92-35.89)	27.84 (26.3-30.2)	**0.03**
**ANP (preop.) [nmol/l]**	6.86 (5.68-8.06)	9,995 (7.53-14.02)	**0.009**

Comparison of individuals converted to SR and remaining in AF at 6 months after HIFU ablation.

According to the multivariate logistic regression analysis among preoperative variables only plasma ANP level was an independent predictor of cardiac rhythm after epicardial HIFU ablation in patients with LsPe AF (Odds Ratio 0.733; Coefficient -0.31; p = 0.03) (Table 3).

**Table 3:** Multivariate logistic regression analysis

	**Cardiac rhythm at 6 months after HIFU ablation**		
**Preop. Parameter**	**Coefficient**	**Odds ratio**	**p value**
LAD (mm)	0.204	1.227	0.135
LA area (cm^2^)	-0.0174	0.983	0.90
ANP (nmol/l)	-0.310	0.733	**0.03**

Associations between preoperative clinical parameters in longstanding persistent AF and cardiac rhythm according multivariate logistic regression analysis.

**Figure 5:**
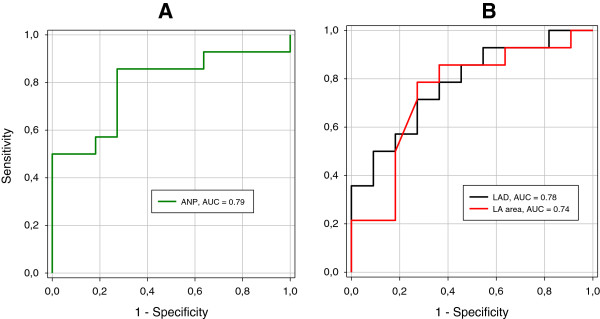
**ROC curves for predictors of outcome after epicardial HIFU ablation. (A)** ROC curve for preoperative ANP level and **(B)** ROC curves for LAD and LA area in long standing persistent AF. Baseline ANP level is the most useful predictor of heart rhythm at 6 months after HIFU ablation in longstanding persistent AF; ROC: receiver operating characteristic; ANP: atrial natriuretic peptide; LAD: left atrial diameter; LA: left atrium; AUC- area under the curve.

Finally, to test the performance of the predictors, we performed ROC curve analysis for baseline ANP levels, LAD and LA area. We found that the area under the ROC curve (AUC) for baseline ANP levels was 0.79 (p = 0.013), for LAD 0.78 (p = 0.018) and for LA area 0.74 (p = 0.04). These findings indicate that, the preoperative ANP levels demonstrate high quality and power as predictor of outcome after epicardial HIFU ablation among patients with LsPe AF. Furthermore, preoperative plasma ANP was also more sensitive and had better positive predictive value (PPV) in prediction of SR after ablation (85.7% sensitivity, 80% PPV and 72,7% specificity for ANP at cut-off value 7.5 nmol/l) than the classic echocardiographic parameters associated with LA size (71.4% sensitivity, 76,9% PPV and 72,7% specificity for LAD and LA area at cut-off 50 mm and 29 cm2, respectively) (Figure 5).

In AF ablation group among patients with LsPe AF, 80% of individuals with a preoperative ANP level greater than 7.5 nmol/l presented with SR at 6 months follow-up. In contrast, only 20% of individuals with a baseline ANP level below 7.5 nmol/l successfully converted to SR after ablation (p = 0.005) (Figure 2). In patients with Pa and Pe AF and baseline ANP levels >7.5 nmol/l we observed stable SR at 6 months in 100% of cases, as compared to 88% of patients with SR in the group with ANP levels <7.5 nmol/l, which however was not statistically significant.

## Discussion

Our study is the first - to our knowledge - to evaluate the relationship between a biochemical marker, echocardiographic characteristics and heart rhythm in 6 months follow-up after epicardial HIFU ablation in patients with concomitant AF undergoing cardiac surgery. Our results indicate among patients with LsPe AF significantly higher baseline ANP levels in individuals with successful conversion to SR at 6 months follow-up after HIFU ablation compared to patients, who remained in AF despite ablation, suggesting that plasma ANP levels may be a noninvasive biochemical predictor of success after HIFU ablation.

In addition, in LsPe AF, which revealed the lowest conversion rate to SR among all types of AF [8] (in our population 56% of SR after 6 months), patients with baseline ANP levels > 7.5 nmol/l had high success rate of SR restoration (80.0% after 6 months). In contrast in patients with baseline ANP levels < 7.5 nmol/l, the success rate was significantly lower (20.0% after 6 months, p < 0.005). A similar relationship between preoperative ANP levels and effectiveness of ablation by maze procedure was also presented by Yoshihara et al. [15]. Sato et al. showed a strong association between ANP-to-BNP ratio and rhythm after the maze procedure in patients with mitral valve disease [19]. Furthermore our results indicate that among preoperative parameters only baseline plasma ANP levels are independent predictors of cardiac rhythm after HIFU ablation in patients with LsPe AF. Numerous studies reported that the patients with successful SR restoration after classic maze procedure had significantly smaller LAD as determined by preoperative echocardiography, than those who remained in AF [19-21]. In our study, preoperative LAD with cut-off at 50 mm was a dependent predictor of success after ablation, which is consistent with previous reports [11,12]. In addition, we demonstrate that the patients with LsPe AF those remained in AF despite HIFU ablation had not only larger left atrial size (LAD >50 mm and LA area >29 cm^2^) but also showed significantly lower preoperative plasma ANP levels, compared to those who successfully converted to SR at 6 months. Furthermore our study indicates that baseline plasma ANP levels in LsPe AF are more useful for predicting the outcome of HIFU ablation than conventional echocardiographic characteristics, such as LAD and LA area.

We discovered an increase of plasma ANP level after epicardial HIFU ablation, which is contrary to other studies which described dramatically decreased ANP levels after classic maze procedure (CMP) [17]. Decreased ANP levels are the main cause for acute postoperative fluid retention with pulmonary oedema and symptomatic pleural effusion observed in 12-36% of patients after CMP. On the other hand, higher ANP levels are assumed to improve kidney function and prevent renal insufficiency after cardiac surgery [21], have beneficial impact on pulmonary pressure reduction [22] and protect against ischemia and reperfusion injury [23]. It is believed that amputation of both atrial appendages in the original Cox-maze procedure is a major cause of postoperative ANP decrease [17]. Therefore, according to Yoshihara et al. preservation of RAA in the modified maze procedure effectively protects against postoperative decrease of ANP and it has beneficial impact on the postoperative course [21]. Hypothetically, an elevated ANP level after HIFU ablation may have a beneficial effect on postoperative course, but this question requires further investigations. In our study we amputated RAA in 100% and LAA in 21.4% of cases, therefore the increased ANP levels on POD 1 must be associated with other factors than preservation of atrial auricles. Since increased ANP levels from POD 7 to 3 months in follow-up were also observed in the control group with SR, we suspect that immediate postoperative ANP increase is rather a reaction to surgical trauma, perioperative higher volume load and atrial stretch. Other studies revealed also similar increase of ANP levels observed on POD 1 and 2 after CABG [24]. Surprisingly, cardiopulmonary bypass and its duration did not influence plasma ANP levels significantly [25]. Among patients with AF, we observed a significant increase in postoperative ANP levels only in the ablation group, but not in the control group without ablation. We might hypothesize that higher ANP concentrations could also be derived from necrotizing wall injury due to ablation. Nevertheless, among the individuals who did not undergo HIFU ablation, we observed that patients in the SR control group had a much greater increase in ANP levels on POD 1 than patients in the AF control group without ablation, (83% vs 24% increase from baseline level). This might suggest an impaired capability of the atria to release ANP, when they are fibrillating. Systemic ANP level depends not only on atrial stretching secondary to hemodynamic changes, but also on structural state and remodeling of atria. Previous study suggested that plasma ANP levels in patients with AF reflected degenerative changes and inversely correlated with the level of LA fibrosis [15]. Probably because the degenerative changes are most expressed in LsPe AF, only in these patients preoperative plasma ANP levels were independent predictors of cardiac rhythm after ablation in the present study. While patients with Pa and Pe AF with intermittent burden of atrial wall-stretch are heterogeneous as for factors stimulating ANP release. It might explain, why in our population we found the lowest basal plasma ANP levels in the SR control group, increased in patients with paroxysmal and persistent and highest in patients with longstanding persistent AF. This data partially confirmed the findings by Cao, which showed elevated plasma ANP levels in paroxysmal and persistent AF compared to SR group [5].

**Figure 6:**
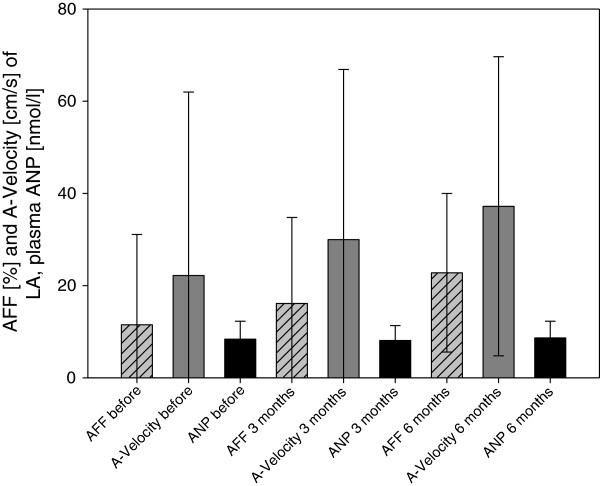
**ANP concentrations, AFF and A-velocity of LA after epicardial HIFU ablation.** Significant increase of AFF and A-velocity at 6 months compared to the baseline and no changes of ANP levels in 6 months follow-up. ANP: atrial natriuretic peptide; AFF: atrial filling fraction; LA: left atrium.

We present the impact of HIFU ablation on atrial hemodynamic active function, using echocardiographic characteristics such as AFF and velocity of A-wave in 6 months follow-up. The most important purpose of surgical AF treatment is restoration of coordinated atrial contraction and their transport function resulting in improved cardiac hemodynamics, reduced risk of thromboembolism and stroke [26]. In patients, who had converted to SR at 6 months follow-up we observed active contractility of both atria in 80.8% of patients, of the left atrium in 84.6% and of the right atrium in 88.5%. Previous studies had shown lower rates of recovery of LA contractility in follow-up after ablation despite stable SR, both in only left atrial ablation (70% for cryoablation) [27] and in biatrial ablation (63% for modified radiofrequency Maze procedure) [28] or in CMP (73%) [29]. However, there is no data in the literature on atrial contractility after ablation using HIFU. We found significant increase of both AFF and A wave velocity of the left atrium in 6-months follow-up. These findings support the assumption that left “atrial stunning” after epicardial HIFU ablation last about 6 months and anticoagulation is necessary throughout this time. We found no correlation between recovery of LA contractility and ANP level alteration (Figure 6). We suspect that the described decreased ANP levels associated with SR restoration and recovery of atrial contractility [30,31] are masked by additional ANP release due to surgical trauma, necrosis in ablated tissue or higher volume overload in the early postoperative period.

68.4% of our entire study population achieved restoration of SR within 6 months. This corresponds with studies published recently (62-66% conversion rate) [11,12]. However, our success rate was lower than the first reports on HIFU-ablation (83.2%-85%) [8,10]. We explain this by a lower percentage of individuals with Pa AF in our study population (11.9%) compared to the study of Groh et al. (33.3%) [10] and lower proportion of patients with concomitant mitral valve surgery (23.8%) in comparison to the study conducted by Ninet et al. (44.6%) [8]. It is well-known that the success rate of ablation is significantly higher in individuals with Pa AF compared to those with LsPe and permanent AF, and correction of mitral valve disease have a beneficial effect on SR restoration [26]. We did not perform any positive selection of patients regarding predictors of successful ablation. In reality every patient with AF, who required surgical treatment of underlying heart disease, has undergone concomitant ablation. This may result in the higher proportion of LsPe and lower of Pa AF in our study.

Epicardial approach allows surgeons to replicate mini-maze lesions on beating heart. It requires controlled propagation of energy without risk of heart and collateral structures damage or coagulates formation in circulating blood. For this reason only the systems based on RF, microwave and HIFU are suitable, whereas laser and cryoablation can be used exclusively from endocardial. Thickness of left atrial wall and epicardial fat layer [32] and the “heat-sink effect” of circulating blood volume are the main factors reducing transmurality and thereby outcome of epicardial methods. Effectiveness of epicardial ablations in restoring of SR by the same lines concept are comparable and ranged between 62.2-65% for microwave [33], 62-85% for HIFU [9,12] and 67,5-82% for RF [34,35] at different follow-up periods. Following the acquisition of Guidant company, production of Flex 4 and 10 sole devices using microwave was stopped. Presently, the SJM company closed for business reasons the branch of Epicor production in order to focus on new market of transcatheter aortic valves technologies. As a consequence actually only RF remains on the market for epicardial approach.

### Limitations

Our study population was relatively small with a patient panel of 78 individuals, thereof 42 in the ablation group. Nevertheless, the average number of patients included in other studies [15,17,19,21,36] evaluating ANP and surgical ablation was 37, ranging from 19 to 62 patients. Our study was neither randomized controlled nor double-blinded due to an intention to offer ablation to every patient affected by AF. Therefore recruitment to the AF control group without ablation was very difficult. Finally our AF control group without ablation included only these patients, in whom we found intraoperative obstacles to safely perform the procedure which limited its size (n = 16) and contributed to slightly higher risk in Euroscore II in this group, as compared to the AF ablation group.

## Conclusion

(1) Our results indicate that preoperative ANP levels may be a new biochemical predictor of successful surgical HIFU ablation in patients with concomitant LsPe AF. (2) Surgical HIFU ablation of concomitant AF causes a significantly improvement of atrial mechanical function and gradual increase of AFF and did not associate with alteration of atrial endocrine secretion at 6 months follow-up. (3) Our results indicate that epicardial HIFU causes an increased secretion of plasma ANP on POD 1. Since drop of ANP after maze ablation is the main cause of severe fluid retention, an ANP increase after HIFU might have a beneficial impact on postoperative course; however, further study is required to resolve this question.

## Abbreviations

AF: Atrial fibrillation; Pa: Paroxysmal; Pe: Persistent; LsPe: Longstanding persistent; ANP: Atrial natriuretic peptide; AFF: Atrial filling fraction; CMP: Cox-maze procedure; CABG: Coronary artery bypass grafting; HIFU: High intensity focused ultrasound; LA: Left atrium; LAD: Left atrial diameter (maximal); LA area: Left atrial area (maximal); LAA: Left atrial appendage; RAA: Right atrial appendage; RF: Radiofrequency; SR: Sinus rhythm.

## Competing interest

The authors declare that they have no competing interests.

## Authors’ contributions

MP developed study protocol, carried out follow up’s, obtained data, analyzed data and wrote the manuscript. RF developed study protocol, coordinated the study and provided critical revision of the manuscript. MP carried out the immunoassay and helped to perform the statistical analysis. MF helped to draft the manuscript. FN and MW coordinated the study. All authors read and approved the final manuscript.
